# Management einer mehrfragmentären Glenoidfraktur: Fallbericht eines zweizeitigen Operationsverfahrens

**DOI:** 10.1007/s00113-022-01196-7

**Published:** 2022-07-14

**Authors:** Oliver Tenfelde, Andreas Karwatzke, Paola Kappel, Maurice Balke, Arasch Wafaisade

**Affiliations:** 1 Lehrstuhl für Unfallchirurgie, Orthopädie und Sporttraumatologie, Universitätsklinik Witten/Herdecke am Klinikum Köln-Merheim, Ostmerheimer Str. 200, 51109 Köln, Deutschland; 2 Sportsclinic Cologne, Universität Witten/Herdecke, Ostmerheimer Str. 200, 51109 Köln, Deutschland

**Keywords:** Schultergelenk, Schulterpfannenbruch, Schulterluxation, Zwei-Pfeiler-Frakturen, Knöcherne Bankart-Läsion, Ideberg‑Typ 6, Shoulder joint, Glenoid fracture, Shoulder dislocation, Two-pillar fractures, Bony Bankart lesion, Ideberg type 6

## Abstract

Es wird der Fall eines 65-jährigen Patienten geschildert, welcher sich nach einem Sturz aus 2 m Höhe eine mehrfragmentäre Glenoidfraktur zuzog. Die Krafteinwirkung auf den angelegten Arm führte zur vollständigen y‑förmigen Gelenkdestruktion mit großem posteroinferioren und einem großen anteroinferioren Glenoidfragment. Die Operation bestand in einer 2‑zeitigen Technik aus offenem und arthroskopischem Verfahren. So folgte zunächst die offene Osteosynthese eines Fragmentes von dorsal mittels Schraubenosteosynthese. Vier Wochen später wurde die arthroskopische Refixation des anterioren Glenoidfragmentes im Sinne einer knöchernen Bankart-Läsion mittels Zielinstrumentarium und Endobutton®-Fixation durchgeführt.

Mehrfragmentäre Glenoidfrakturen des Ideberg-Typs 6 sind selten und sollten insbesondere bei großer Gelenkstufe, Instabilität im Glenohumeralgelenk und jüngeren Patienten operativ versorgt werden. Bei fehlender Handlungsempfehlung in der Literatur sind individuelle Operationsstrategien erforderlich.

## Abstract (en)

This article describes a multifragmentary glenoid fracture in a 65-year-old patient after falling from a height of 2 m. The accident led to complete Y‑shaped destruction of the glenohumeral joint with a large posteroinferior fragment and a large anteroinferior glenoid fragment. The treatment consisted of a two-stage surgical procedure and a combination of open and arthroscopic techniques. First, open surgery of one fragment from the dorsal side was performed using screw osteosynthesis. Arthroscopic refixation of the anterior glenoid fragment (bony Bankart lesion) followed 4 weeks later using a guided Endobutton® technique (Smith & Nephew). Ideberg type 6 multifragmentary glenoid fractures are rare and should be treated surgically, especially with dislocated fragments, instability in the glenohumeral joint and in younger patients. Given the lack of evidence in the literature, individual surgical strategies are necessary.

## Anamnese

Ein 65-jähriger Patient wurde in der Notaufnahme vorstellig. Er berichtete, dass er vor 2 Tagen während Gartenarbeiten von einer 2 Meter hohen Astgabel auf den angelegten rechten Oberarm gestürzt war. Er verspürte dabei ein Ausrenken der rechten Schulter. Nach Vorstellung beim niedergelassenen Orthopäden mit Röntgendiagnostik erfolgte die Einweisung bei Verdacht auf eine mehrfragmentäre Glenoidfraktur.

## Klinischer Befund

Inspektorisch zeigte sich ein beginnendes Hämatom über dem M. deltoideus mit prominentem Akromion. Die aktive Abduktion und Außenrotation des Armes waren schmerzfrei nicht möglich. Es zeigte sich eine deltoidale, oberflächliche Schürfwunde bei intakter peripherer Durchblutung und Sensibilität. Es wurde bei Bewegung eine deutliche Krepitation palpiert. Der initiale Constant-Murley-Score betrug 15 Punkte.

## Radiologischer Befund

**Figure 1:**
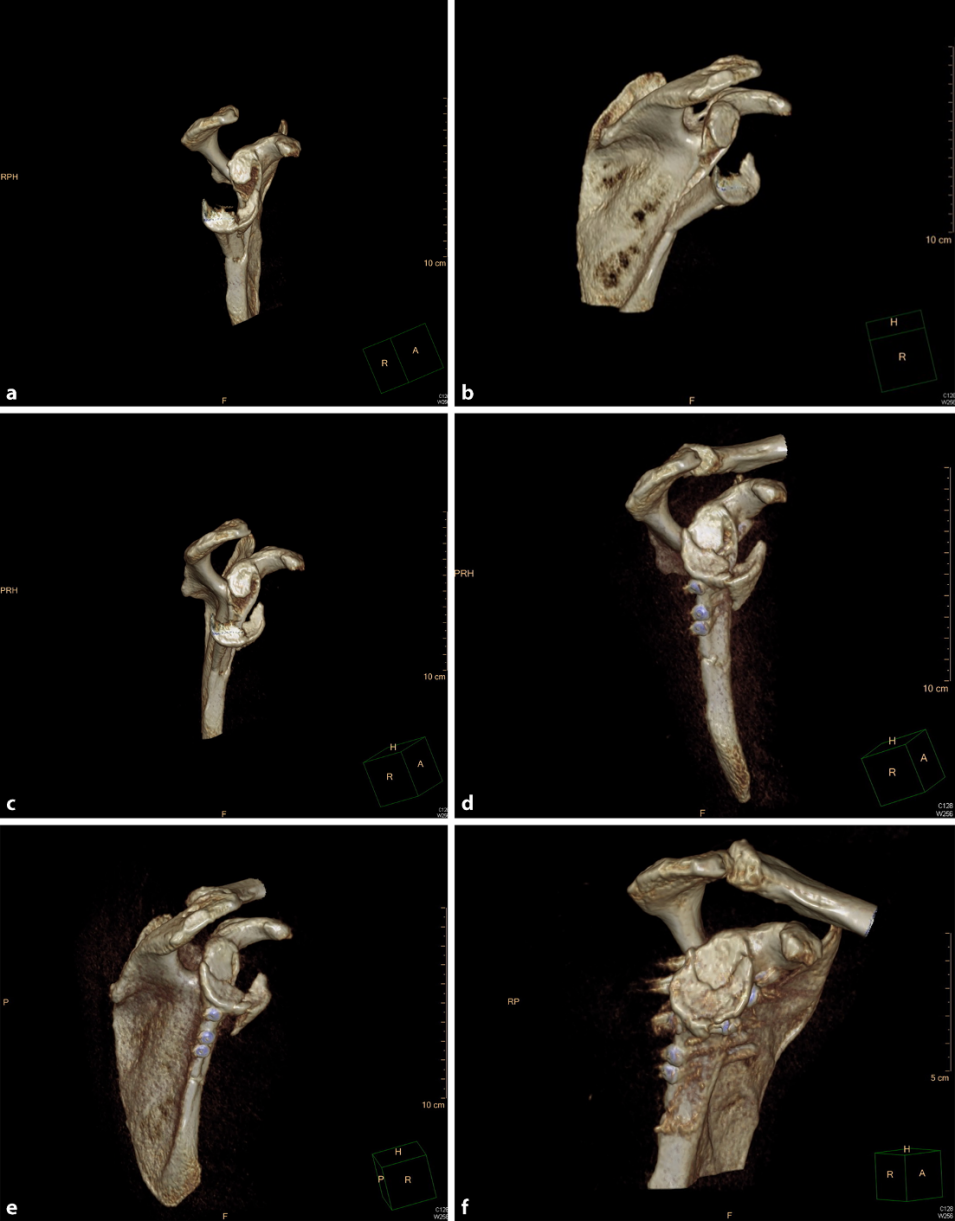

Auf den mitgeführten Röntgenaufnahmen der Schulter in 2 Ebenen sowie der folgenden Computertomographie mit 3D-Rekonstruktion und Subtraktion des Humeruskopfes zeigte sich eine dehiszente Spaltfraktur des Collum scapulae mit Einbezug der Cavitas glenoidalis und Margo lateralis. Der Humeruskopf kam in die Gelenkfläche imprimiert und kraniodorsal subluxiert zur Darstellung. Das Glenoid präsentierte sich Y‑förmig in 3 Fragmente aufgespalten. Ein großes posteriores Fragment mit den Maßen 35 mm × 35 mm und ein weiteres anteroinferiores Fragment von 15 mm × 16 mm waren ersichtlich (Abb. 1a und 2a–c).

**Figure 2:**
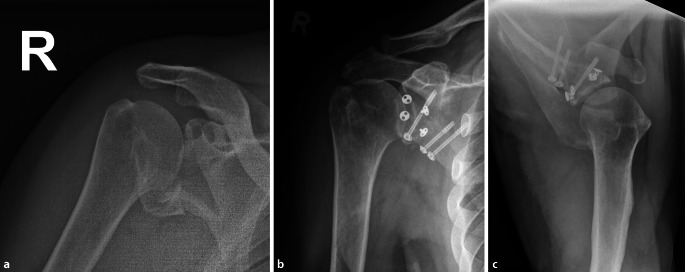

## Therapie

Bei deutlicher Fragmentdislokation größer 5 mm und vollständig destruierter Gelenkfläche wurde die Indikation zur operativen Versorgung gestellt, die 2‑zeitig geplant wurde.

**Figure 3:**
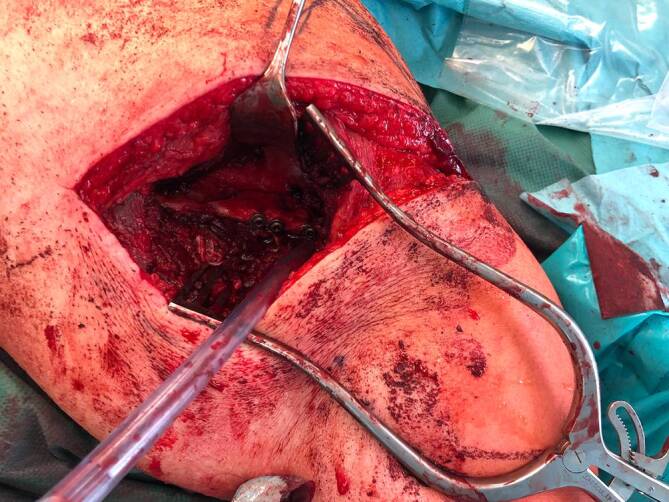

Der erste Eingriff erfolgte in Bauchlagerung nach Allgemeinanästhesie. Mittels modifiziertem Zugang nach Brodsky [2] wurde von der dorsolateralen Akromionecke senkrecht nach kaudal verlaufend präpariert. Der Deltamuskel wurde am Unterrand mittels Kocherhaken aufgeladen (Abb. 3). Nach Eröffnen der Faszie wurden der M. infraspinatus und M. teres minor unter Visualisierung des Endastes des N. axillaris dargestellt. Durch das Intervall der beiden oben genannten Muskeln konnte sodann die Fraktur aufgesucht und reponiert werden. Zunächst erfolgten die Transfixierung mit 3 K-Drähten und die Überbohrung unter Bildwanderkontrolle. Schließlich konnte die Osteosynthese mittels Schrauben (ASNIS 4,0 mm, Fa. Stryker, Kalamazoo, MI, USA) vorgenommen werden. Es folgte ein 4‑wöchiges Intervall mit Ruhigstellung in einer Schulterorthese. Die Nachbehandlung bestand aus passiver Mobilisation bis 60°-Anteversion und Abduktion unter vollständiger Vermeidung der Außenrotation. In der klinischen Verlaufskontrolle zeigte sich das Schultergelenk mit 2fach positiver Schublade noch deutlich instabil. Computertomographisch präsentierte sich eine stufenlose Reposition mit Zeichen der knöchernen Konsolidierung (Abb. 1d–e), sodass der zweite Eingriff in arthroskopischer Technik vorgenommen werden konnte. Hierbei kamen ein instabiler Bizepssehnenanker im Sinne einer SLAP-Läsion Typ 2 nach Snyder und eine intakte Rotatorenmanschette zur Darstellung. Es wurde mit der Mobilisation des knöchernen Fragmentes vom Skapulahals begonnen. Mittels von dorsal eingebrachtem Zielinstrumentarium [7] konnte die Fraktur zunächst mit über das Zielgerät von dorsal eingebrachten kanülierten K‑Drähten retiniert und schließlich mittels zweifachem Endobutton®-Konstrukt (Fa. Smith & Nephew, London, UK) fixiert werden. Das konsolidierte posteriore Fragment diente beim Verknoten des Endobutton®-Konstruktes mit 75 Nm Spannung als Widerlager.

Sechs Monate postoperativ zeigte sich eine stabile, schmerzfreie Gelenkführung bei allerdings noch endgradiger Bewegungseinschränkung mit einem Constant-Murley-Score von 69 Punkten. In der Computertomographie kamen alle Fragmente, bei nur minimaler verbliebener Gelenkstufe < 2 mm, knöchern konsolidiert zur Darstellung (Abb. 1f).

## Diskussion

Mehrfragmentäre Glenoidfrakturen, insbesondere der Fossa glenoidalis mit Einstrahlung in die Skapula sind im Vergleich zu Frakturen des glenoidalen Rings selten [8]. Sie sind meist Folge von Hochrasanztraumata und direktem Anprall auf den Humeruskopf [5]. Nach Ideberg lassen sich diese Frakturen typischerweise in 6 Grade einteilen, wobei 3 oder mehr Fragmente Typ 6 entsprechen [4]. Eine detailliertere Klassifikation der Frakturmorphologie wird bei mehreren Fragmenten nicht vorgenommen. Die „Arbeitsgemeinschaft für Osteosynthesefragen“ (AO) bietet eine alternative Einteilung mit hoher Inter- und Intraobserver-Reliabilität, welche jedoch aufgrund der Komplexität klinisch seltener Anwendung findet [3].

Frakturen des vorderen Glenoidrandes (Ideberg-Typ 1a) kommen am häufigsten vor und werden inzwischen standardmäßig arthroskopisch operiert. Sie weisen die breiteste Evidenz in der Literatur auf [5]. Glenoidfrakturen von 2 und mehr Fragmenten, insbesondere solche mit großem dorsalem Frakturanteil, sind technisch anspruchsvoll und werden nur selten minimal-invasiv versorgt. Es existieren lediglich vereinzelt Fallberichte, in denen ein arthroskopisches Vorgehen beschrieben wurde [9]. Alternativ bleibt ein offenes Operationsregime mittels ausgedehnter Arthrotomie von ventral und dorsal, welches ein hohes Weichteiltrauma mit erheblichem Komplikationsrisiko birgt [6]. Neben minimal-invasiven Zugängen ist mittels Arthroskopie eine vollständige Visualisierung des Gelenkes möglich. Des Weiteren können Begleitverletzungen adressiert werden. Offene Zugänge nach Judet oder im Sinne eines deltoideopektoralen Zuganges machen lediglich eine Darstellung von weniger als 50 % der glenoidalen Gelenkfläche möglich. Die superiore Gelenkfläche (zwischen 11:30 Uhr und 01:30 Uhr) wird dabei als nicht darstellbar beschrieben [1].

Durch die Seltenheit der Verletzung und einen Mangel an evidenzbasierten Handlungsempfehlungen in der Literatur sind häufig individuelle Lösungen notwendig. Angelehnt an das operative Prinzip von Zweipfeilerfrakturen des Acetabulums kann ein 2‑zeitiges Vorgehen erfolgen, mit zunächst offener Rekonstruktion der dorsalen Gelenkfläche. Postoperativ verbleibt lediglich ein „knöcherner Bankart“ Ideberg-Typ 1a, was dann eine arthroskopische Versorgung möglich macht. Bislang ist die Kombination dieser Operationstechniken im 2‑zeitigen Vorgehen bei mehrfragmentärer Glenoidfraktur noch nicht in der Literatur beschrieben. Der vorgestellte Patientenfall wies bereits im kurzfristigen Follow-up ein zufriedenstellendes Ergebnis auf.

## Fazit für die Praxis

- Mehrfragmentäre Glenoidfrakturen sind eine Seltenheit und sollten bei jüngeren Patienten, Instabilität im Glenohumeralgelenk und Gelenkstufe > 5 mm operativ versorgt werden.
- Eine rein offene Versorgung ist mit großem ventralen und dorsalen Weichteiltrauma sowie hohem Risiko für postoperative Komplikationen verbunden.
- Insbesondere bei großem dorsalen Fragment ist ein zweizeitiges Vorgehen in Erwägung zu ziehen, bei dem zunächst der dorsale Anteil offen osteosynthetisch fixiert wird. Nach der Konsolidierung kann dann arthroskopisch im Sinne eines knöchernen Bankart-Repair operiert werden.
